# Silyl Anion Initiated Hydroboration of Aldehydes and Ketones

**DOI:** 10.1002/chem.202000897

**Published:** 2020-07-02

**Authors:** Martin W. Stanford, Alessandro Bismuto, Michael J. Cowley

**Affiliations:** ^1^ School of Chemistry University of Edinburgh Joseph Black Building, David Brewster Road Edinburgh EH9, 3FJ United Kingdom

**Keywords:** carbonyl, hydroboration, reduction, silicon, silyl anion

## Abstract

Hydroboration is an emerging method for mild and selective reduction of carbonyl compounds. Typically, transition‐metal or reactive main‐group hydride catalysts are used in conjunction with a mild reductant such as pinacolborane. The reactivity of the main‐group catalysts is a consequence of the nucleophilicity of their hydride ligands. Silicon hydrides are significantly less reactive and are therefore not efficient hydroboration catalysts. Here, a readily prepared silyl anion is reported to be an effective initiator for the reduction of aldehydes and ketones requiring mild conditions, low catalyst loadings and with a good substrate scope. The silyl anion it is shown to activate HBpin to generate a reactive borohydride in situ which reacts with aldehydes and ketones to afford the hydroboration product.

## Graphical abstract

**Hydroboration catalysts** based on environmentally benign and earth abundant main group compounds offer high potential as an alternative to transition‐metal catalysts. Silicon is abundant and has no inherent toxicity, but has found limited use in hydroboration catalysis. In this work, it is shown that a silyl anion is an effective initiator for the hydroboration of carbonyl compounds requiring low catalyst loadings and mild conditions and with good functional group tolerance.

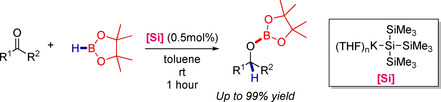

The reduction of aldehydes and ketones is an important organic transformation. Recently, there has been a drive for the discovery of new catalytic processes exploiting mild and easy to handle reducing agents, such as pinacolborane (HBpin). A large number of transition‐metal and main‐group catalysts have already been reported to catalyze the hydroboration of carbonyls using reducing agents including HBpin.^1^ In particular, main‐group metal hydride catalysts have been widely explored for the hydroboration of carbonyl compounds,1b, 1c, 2 including the seminal work of Hill using a magnesium hydride,2b the low oxidation state germanium and tin hydrides reported by Jones,2c and aluminum catalysts reported by Roesky,2d Inoue2e and us.2g In most instances, these catalysts were reported to hydrometalate the substrate, followed by metathesis with the borane source to give the boronic ester product and regenerate the metal hydride (Scheme 1).

**Scheme 1:**
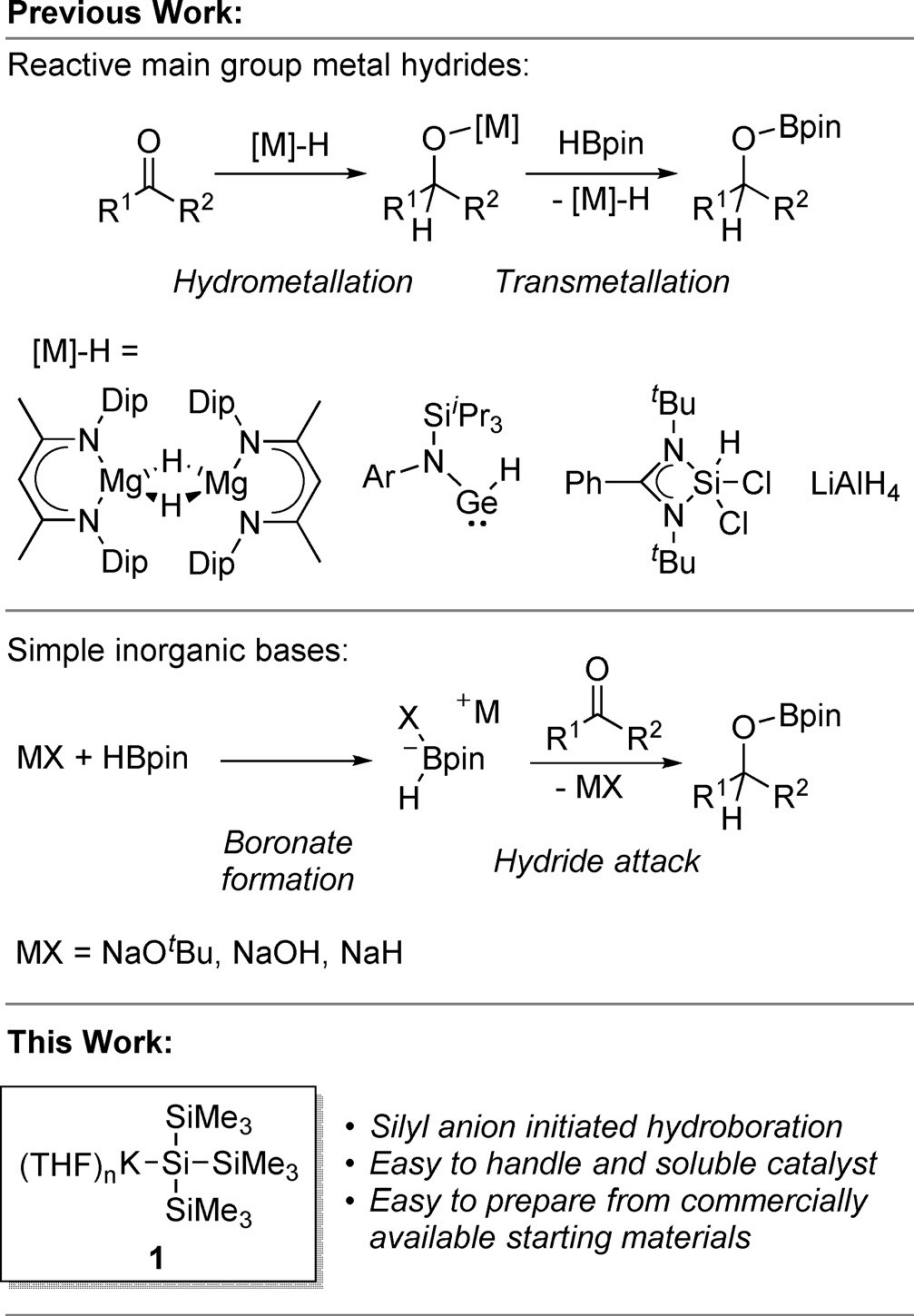
Previously reported main‐group catalysts and mechanisms for the hydroboration of carbonyl compounds.

In contrast to s‐ and p‐block metal hydrides, silicon hydrides are typically less reactive due to the lower electronegativity differences between Si and H.^3^ However, a five‐coordinate amidinato silane has been reported to catalyze the hydroboration of aldehydes.2h Importantly, the catalyst‐free hydroboration of aldehydes has also been reported recently,^4^ demonstrating that HBpin can reduce activated substrates without the use of a catalyst. Thus, future work should focus on more sterically and electronically demanding carbonyl compounds, such as ketones and esters. We wanted to investigate if there was an alternative method to engage silicon catalysts in the hydroboration of such compounds.

Based on recent reports of inorganic bases as pre‐catalysts for hydroboration,^5^, ^6^ we wondered whether a nucleophilic silicon center would result in an active species for hydroboration catalysis. We therefore decided to investigate the common silyl anion, or silicate, KSi(SiMe_3_)_3_, **1**,^7^ as a potential pre‐catalyst. Here, we report that **1** is an effective initiator for the hydroboration of aldehydes and ketones, requiring mild conditions and low catalyst loadings and with a good functional group tolerance (Scheme 2).

**Scheme 2:**
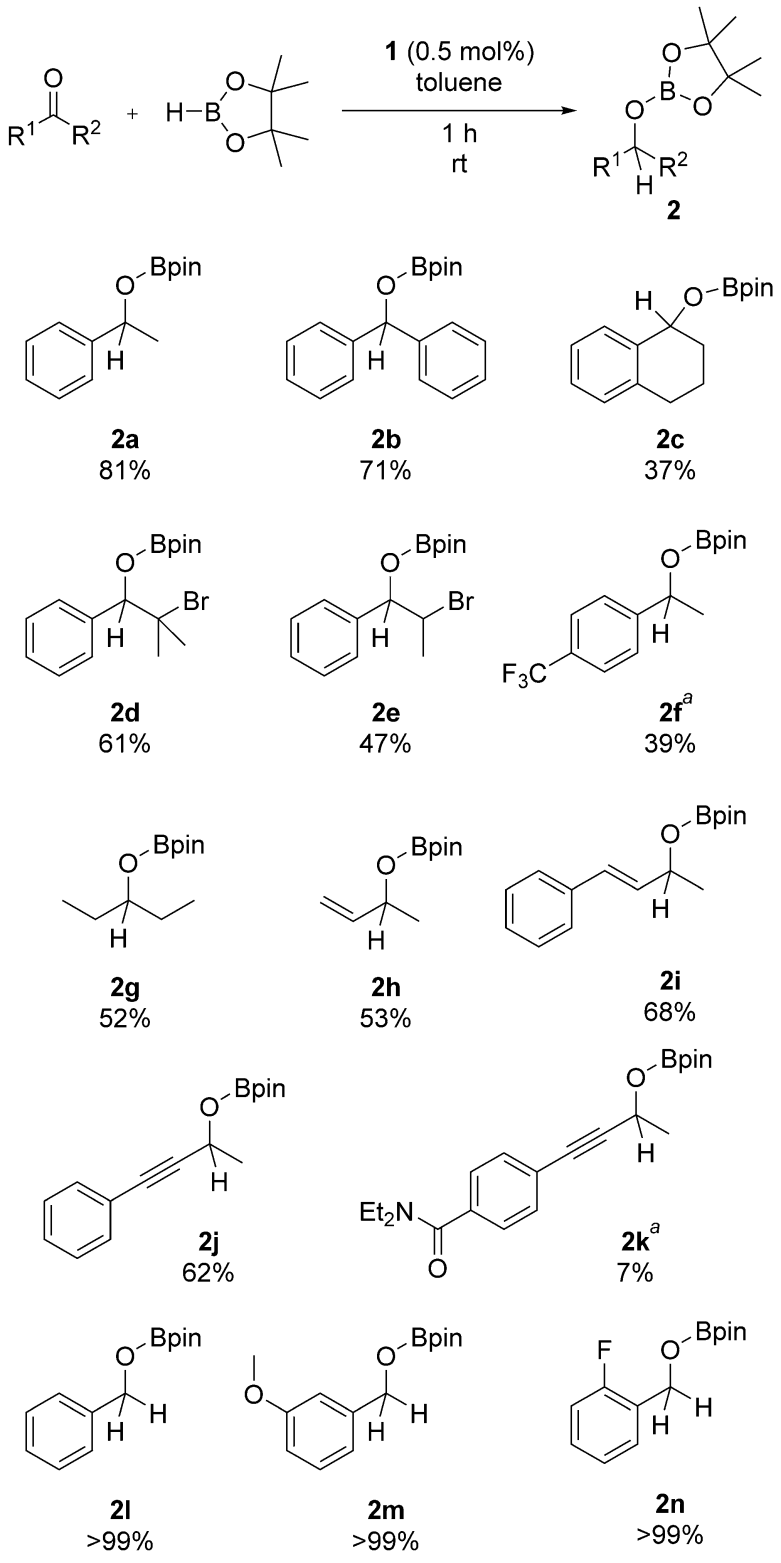
Substrate scope for the silyl anion initiated hydroboration of aldehydes and ketones. Reaction conditions: 0.625 mmol substrate, 0.625 mmol HBpin, 0.5 mol % **1**, 40 μL toluene, room temperature, 1 hour. [a] An additional 160 μL was toluene added in order to dissolve the substrate. NMR yields were measured by ^1^H NMR spectroscopy using 1,3,5‐trimethoxybenzene as an internal standard.

Initially, we tested the hydroboration of acetophenone using HBpin and a substoichiometric amount (3 mol %) of silyl anion **1**. Under solvent free conditions, the boronic ester product **2 a** was afforded in quantitative yields after just twenty minutes at room temperature.

After optimization of the reaction conditions (see Supporting Information), all further reactions were carried out with 0.5 mol % catalyst loading in toluene. Reactions were run for 1 hour at room temperature before quenching with CDCl_3_ in air. Notably, when the catalyst loading was lowered to 0.1 mol %, we were still able to achieve a yield of 75 % after 48 hours, a turnover number of 750. For comparison, the hydroboration of acetophenone by HBpin using KO*t*Bu as an initiator required 5 mol % catalyst loading to achieve 93 % yield after 2.5 hours.^5^

With optimized conditions established, we investigated the scope and functional group tolerance of our procedure. Acetophenone derivatives were successfully converted (**2 a**–**2 f**). Halogen substituents (**2 d** and **2 e**) were tolerated, showing the potential for further functionalization. Vinyl and propargylic ketones were chemoselective for reduction of the carbonyl group (**2 h**–**2 k**). Aldehydes were quantitatively converted to the boronic esters (**2 l**–**2 n**).

We propose a mechanism for the silyl anion initiated hydroboration of carbonyls in Scheme 3, which we base on that previously reported for the alkoxide initiated hydroboration of carbonyls.^5^ The initiation step is coordination of the silyl anion to HBpin to generate borohydride **4**. Delivery of a hydride from **4** to the carbonyl substrate generates an alkoxide intermediate which undergoes further coordination to HBpin, regenerating a borohydride. Coordination of the carbonyl oxygen to the potassium counterion is conceivable, and would accelerate the nucleophilic addition step.1p

**Scheme 3:**
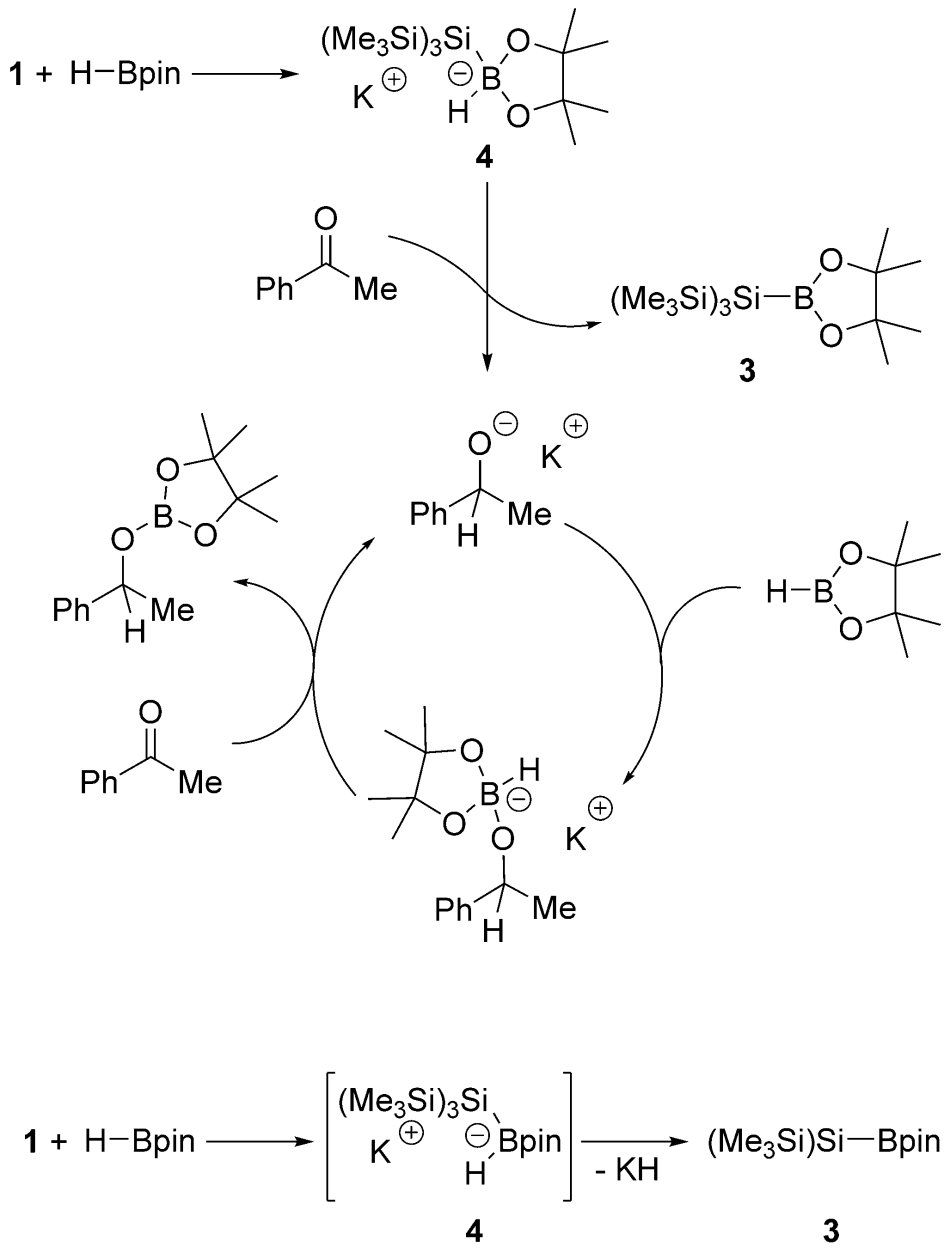
Top: The proposed mechanism for the silyl anion initiated hydroboration of acetophenone. Bottom: Stoichiometric reaction of **1** with HBpin to give silylboronic ester **3**.

We carried out a series of stoichiometric reactions to support the proposed mechanism. Initially, we investigated the stoichiometric reaction of silyl anion **1** with HBpin in C_6_D_6_ and [D_8_]THF. In each case, a gel‐like solid formed immediately. The ^11^B NMR of the C_6_D_6_ reaction mixture shows a single broad singlet resonance at *δ*=37.4 ppm (*ν*_1/2_=230 Hz) which corresponds to silylboronic ester **3**.^8^ In [D_8_]THF, besides the signal for **3** at *δ*=37.4 ppm, a sharp singlet at *δ*=8.4 ppm was observed, which is tentatively assigned to silyl boron‐ate complex **4**, based on similarity with previously reported Na[*t*BuO(H)Bpin] (*δ*=6.2)^5^ and with Brown's trialkoxyborohydrides (*δ* 0.0–6.7).^9^ We also observed, a sharp quartet at *δ*=−45.4 ppm, suggestive of a BH_3_ adduct. The gel‐like nature of the reaction mixture prevented isolation of any of the observed species other than silylboronic ester **3**, which was isolated by filtration of the reaction mixture through silica. The observation of boronic ester **3** and (tentatively) the boronate **4** in stoichiometric reactions supports our proposed mechanism.

It has recently been reported that BH_3_⋅THF can catalyze the hydroboration of alkenes and alkynes.^10^ To rule out silyl‐anion induced decomposition of HBpin to BH_3_, which could act as a catalyst, we carried out a test reaction using 1 mol % BH_3_⋅THF with acetophenone and one equivalent of HBpin under otherwise identical reaction conditions. After one hour at room temperature we only observed trace amounts of the hydroboration product **2 a (**Scheme 4), demonstrating that BH_3_ is not an efficient catalyst under our conditions. This observation is particularly important given the observation of a BH_3_ adduct in the reaction of **1** with HBpin described above. Furthermore, the hydroboration of alkynes did not proceed at room temperature under identical conditions (see Supporting Information).

**Scheme 4:**
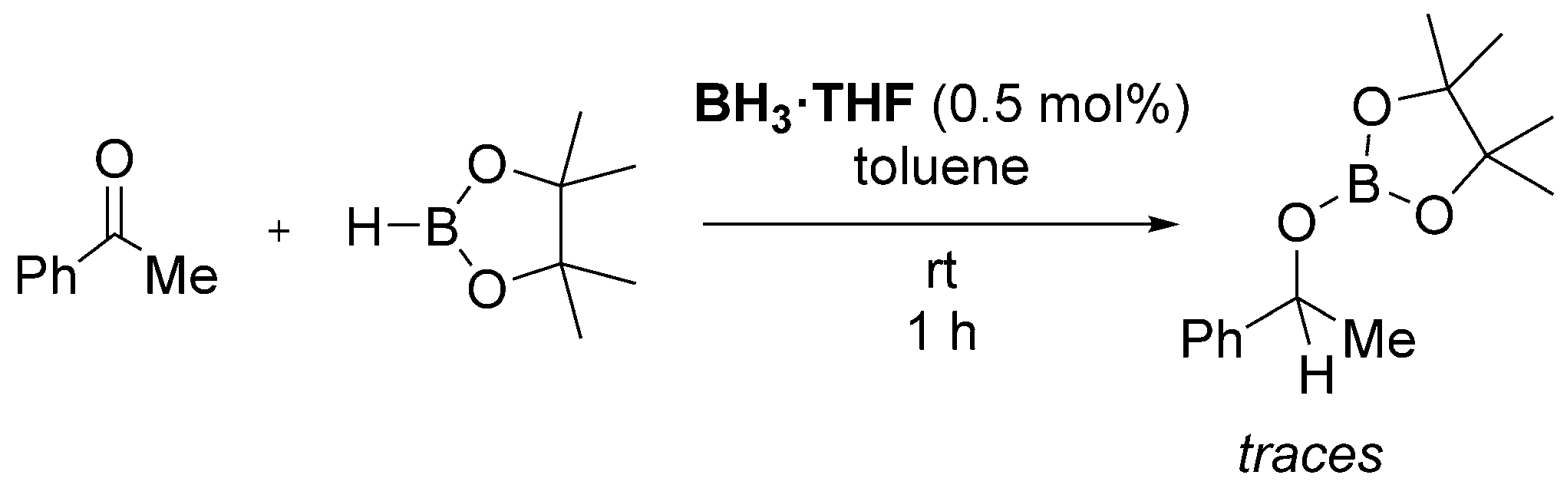
BH_3_⋅THF is not an effective catalyst for the hydroboration of acetophenone.

In conclusion, we have presented a fast, reliable and facile method for the hydroboration of carbonyls using a silyl anion initiator. We have carried out a substrate scope investigation which shows the reaction is tolerant to a range of substituents. Our proposed mechanism is supported by previously reported protocols and stoichiometric studies.

## Conflict of interest

The authors declare no conflict of interest.

## Biographical Information

*Michael Cowley obtained his degree in chemistry at the University of York in the UK, where he also received his PhD in organometallic chemistry in 2008, working with Dr. Jason Lynam. Postdoctoral work followed, first with Simon Duckett on hyperpolarisation techniques for NMR. Later, he joined the group of David Scheschkewitz, at Imperial College and then at the Universität des Saarlandes. He learnt a lot about silicon, and main‐group chemistry, during this time and his enthusiasm for synthetic chemistry was redoubled. In 2013, he moved to the University of Edinburgh to start his own group. In 2016, he was awarded an ERC Starting Grant, and in 2019 he became a Senior Lecturer. He works on fundamental chemistry of the abundant main‐group elements*.

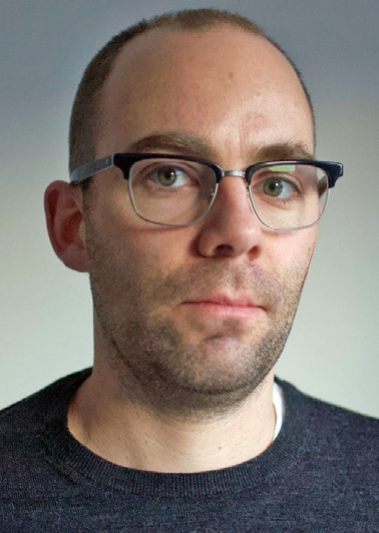

## Supporting information

As a service to our authors and readers, this journal provides supporting information supplied by the authors. Such materials are peer reviewed and may be re‐organized for online delivery, but are not copy‐edited or typeset. Technical support issues arising from supporting information (other than missing files) should be addressed to the authors.

Supplementary
