# Supplementary material for: Silyl Anion Initiated Hydroboration of Aldehydes and Ketones
Source: Chemistry. 2020 Jul 2;26(44):9855–8. doi: 10.1002/chem.202000897 (PMC7496802; doi:10.1002/chem.202000897)
Supplement: Supplementary file 1 — Supplementary [file CHEM-26-9855-s001.pdf]

Chemistry–A European Journal

Supporting Information

Silyl Anion Initiated Hydroboration of Aldehydes and Ketones

Martin W. Stanford, Alessandro Bismuto, and Michael J. Cowley*[a]

Author Contributions

M.S. Conceptualization: Lead; Data curation: Lead; Formal analysis: Lead; Investigation: Lead; Methodology: Lead; Writing - Original Draft: Lead; Writing - Review & Editing: Equal.

Contents

1 General Considerations 2

2 Catalyst Screening 2

Silicon Catalysts 2

BH_3·THF 3

3 Optimisation 3

General Procedure 3

Solvent 4

Stoichiometry 5

Reaction Time 5

4 Substrate Scope 6

General Procedure for the Hydroboration of Aldehydes and Ketones 6

^{1}H NMR Data for Ketone and Aldehyde Hydroboration Products 6

5 Stoichiometric Reactivity 14

Stoichiometric Reaction of HBpin and Silyl Anion 1 14

Preparation of Silyl Boronic Ester 3 *via* Boron-ate 4 14

6 Attempted Hydroboration of Alkynes 15

7 References 16

1 General Considerations

All manipulations were carried out under a dry argon or nitrogen atmosphere using standard Schlenk or glovebox techniques. Solvents were obtained from an Inert solvent purification system and stored over 4 Å molecular sieves. C_6D_6 was dried over potassium then distilled under argon and stored over 4 Å molecular sieves. $CDCl_3$ was used as supplied. ^1H and ^{13}C NMR spectra were recorded on Bruker AVA 400, 500 or 600 MHz spectrometers. ^{11}B NMR spectra were recorded on a Bruker PRO 500 spectrometer. ^1H and ^{13}C spectra were referenced to residual solvent signals.[1] ^{11}B spectra were referenced to $BF_3{\cdot}Et_2O$ as an external standard. Pinacolborane (HBpin) was distilled prior to use to remove traces of BH_3, boric acid and B_2pin_3 which was confirmed by ^1H and ^{11}B NMR. Acetophenone was stored over 4 Å molecular sieves. All other reagents were used as supplied. Catalysts **1**,[2] **A**[3] and **B**[4] were prepared according to literature procedures.

2 Catalyst Screening

Silicon Catalysts

Silicon hydrides with Cp* substituents were investigated as catalysts for the hydroboration of acetophenone. Triethylsilane was also investigated for comparison.

The control reaction (no catalyst) and the reaction using a catalytic amount of triethyl silane only showed trace yields. Silanes **A** and **B** gave 8% and trace yields, respectively. Silyl anion **1** gave **2a** quantitatively under these conditions.

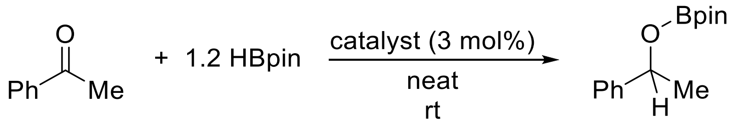

2a

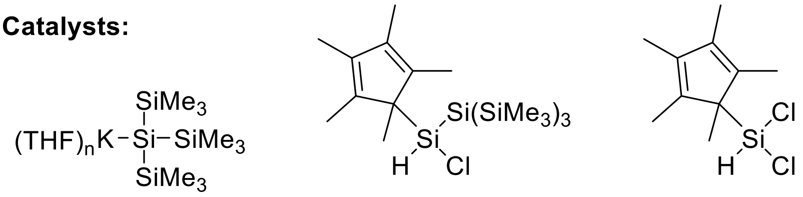

1 **A** **B**

Catalyst	Conversion (%)
**1**	100
**A**	8
**B**	trace
Et_3SiH	trace
None	trace

0.25 mmol acetophenone, 1.2 eq. HBpin, 3 mol% catalyst loading, rt, neat, 20 mins. NMR conversion.

BH_3·THF

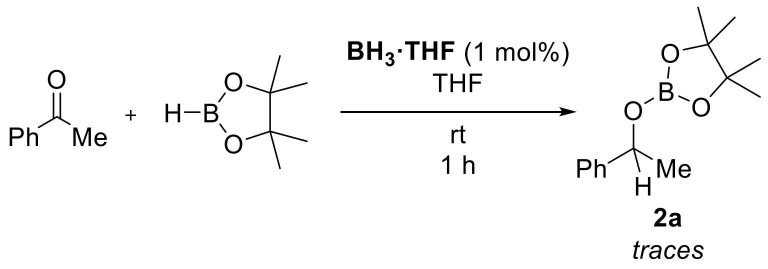

Under an atmosphere of dry nitrogen, pinacol borane (HBpin) (145 µL, 1 mmol) was added to a 1 M solution of BH_3·THF in THF (10 µL, 0.01 mmol). Acetophenone (127 µL, 1mmol) was added and the reaction was left for 1 hour at room temperature. Deuterated chloroform (1 mL) was added in air to quench the reaction. Only trace amounts of hydroboration product **2a** were observed by ^{1}H NMR (<5% conversion).

3 Optimisation

General Procedure

Pinacolborane was added to a solution of anion **1** or crystalline **1** resulting in a colourless solution. Acetophenone was added and the reaction stirred at room temperature for the specified time. $CDCl_3$ was added in air to quench the reactions. For NMR yields, a solution of 1,3,5-trimethoxybenzene in $CDCl_3$ (0.1 M) was added in air to quench the reactions.

Table S1 *– The hydroboration of acetophenone with HBpin catalyzed by silyl anion* ***1*** *at various catalyst loadings.*

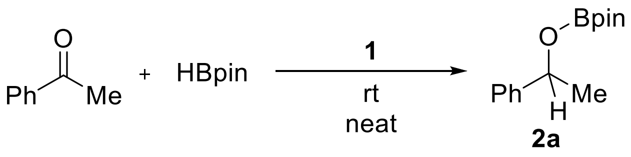

Entry	Catalyst loading (mol%)	Time	Conversion (%)
**1**	3	20 min	100[a]
**2**	1	20 min	67[b]
**3**	0.1	20 min	18[c]
**4**	0.1	1 h	31[c]
**5**	0.1	18 h	60[c]
**6**	0.1	48 h	75[c]

Reaction conditions: [a] 0.3 mmol acetophenone, 0.35 mmol HBpin, room temperature, no solvent; [b] 0.6 mmol acetophenone, 0.7 mmol HBpin, room temperature, no solvent; [c] 72 mmol acetophenone, 84 mmol HBpin, room temperature, no solvent. NMR Conversion.

Solvent

A key advantage of pre catalyst **1** over reported inorganic bases is its solubility. The following solvents were screened at 3 mol% catalyst loading.

Table S2 *– The silyl anion catalyzed hydroboration of acetophenone with pinacolborane in various solvents.*

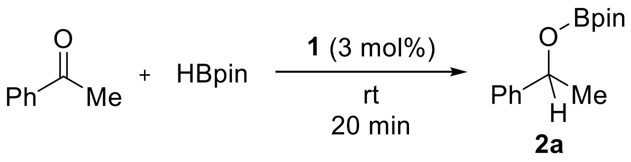

Entry	Solvent	Yield (%)
**1**	None	100
**2**	Toluene	97
**3**	THF	93
**4**	Hexane	89
**5**	DME	61

Yields calculated against 1,3,5-trimethoxybenzene as an internal standard. Reaction conditions: 0.625 mmol acetophenone, 0.656 mmol HBpin, 3 mol% catalyst loading, 0.2 mL solvent, 20 minutes, room temperature.

Toluene and THF were also tested at lower catalyst loadings (Table S3)

Table S3 *– The hydroboration of acetophenone with HBpin catalyzed by* ***1*** *at various catalysts loadings in toluene and THF.*

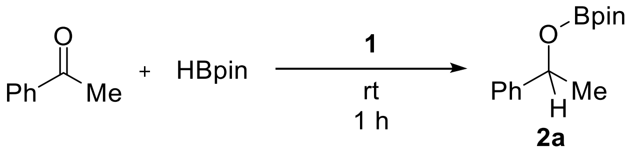

Entry	Catalyst Loading (mol%)	Yield (%)	
		**THF**	**Toluene**
**1**	0.5	75[a]	81[a]
**2**	0.1	60[a]	32[a]
**3**	0.05		29[b]

Reaction conditions: 1 h, room temperature; [a] 0.625 mmol acetophenone and HBpin, 0.1 mL solvent; [b] 1.25 mmol acetophenone and HBpin, 0.2 mL solvent. Yields calculated against 1,3,5-trimethoxybenzene as an internal standard.

Stoichiometry

Table S4 *– The hydroboration of acetophenone catalyzed by silyl anion* ***1*** *using excess HBpin or acetophenone.*

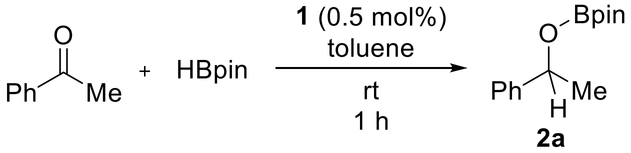

Entry	HBpin eq.	Acetophenone eq.	Yield (%)
**1**	1	1	81
**2**	1.2	1	71
**3**	1	1.2	85

Reaction conditions: 0.625 mmol, room temperature, 40 µL toluene, 0.5 mol% catalyst loading, 1 hour. Yields calculated against 1,3,5-trimethoxybenzene as an internal standard.

Reaction Time

Table S5 *– The yield of the silyl anion catalyzed hydroboration of acetophenone after given time periods.*

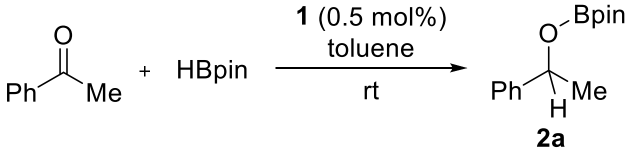

Entry	Time (min)	Yield (%)
**1**	15	80
**2**	30	83
**3**	45	81
**4**	60	81
**5**	90	83

NMR yield measured against trimethoxybenzene as an internal standard. Reaction conditions: 0.625 mmol acetophenone and HBpin, 0.5 mol% catalyst loading, ambient temperature, 1 h. Yields calculated against 1,3,5-trimethoxybenzene as an internal standard.

4 Substrate Scope

General Procedure for the Hydroboration of Aldehydes and Ketones

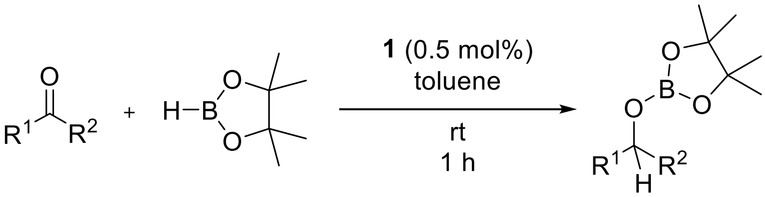

2

Pinacolborane (91 µL, 0.625 mmol) was added to a solution of anion **1** (1.24 mg, 0.0313 mmol) in toluene (20 µL) at room temperature resulting in a colour change from yellow to colourless. The substrate (0.625 mmol) was added and the reaction stirred for one hour at room temperature. After one hour a solution of trimethoxybenzene in $CDCl_3$ was added to the reaction mixture to quench the reaction. Yields calculated against 1,3,5-trimethoxybenzene as an internal standard.

^{1}H NMR Data for Ketone and Aldehyde Hydroboration Products

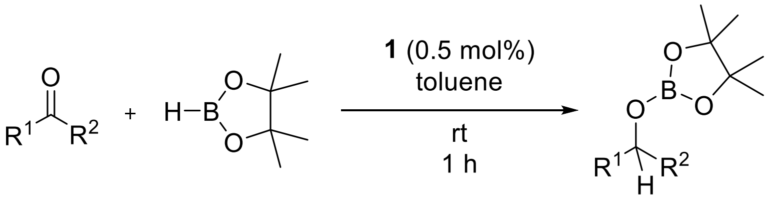

2

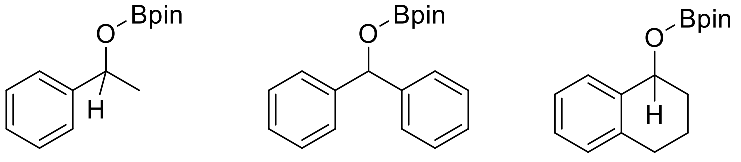

2a 81% | **2b** 71% | **2c** 37%

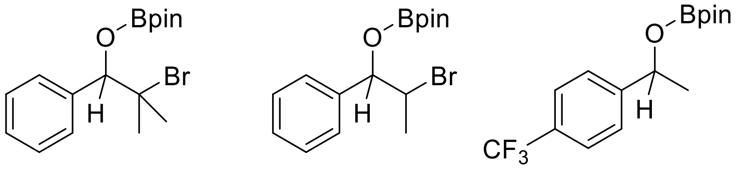

2d 61% | **2e** 47% | **2f**[a] 39%

*For **2a, 2e, 2f** and **2i** analytical data were in accordance with the literature.*[5–7] *Products **2g** and **2h** are previously unreported compounds.* [a] *Extra toluene (200 µL) was added to dissolve the substrate.*

2a (400 MHz, $CDCl_3$, 300 K): δ (ppm) 7.39-7.37 (m, 2H, Ar-*H*), 7.34-7.31 (m, 2H, Ar-*H*), 7.26-7.23 (m, 1H, Ar-*H*), 5.24 (q, 1H, $^3J_{H-H}$ = 6.5 Hz, C-*H*), 1.49 (d, 3H, $^3J_{H-H}$ = 6.5 Hz, Me), 1.23 (s, 6H, Bpin-Me), 1.20 (s, 6H, Bpin-Me).[5]

Yield (81%) calculated against 1,3,5-trimethoxybenzene (19.3 mol%): δ 6.08 (s, 3H, Ar-*H*), 3.74 (s, 9H, O-CH_3).

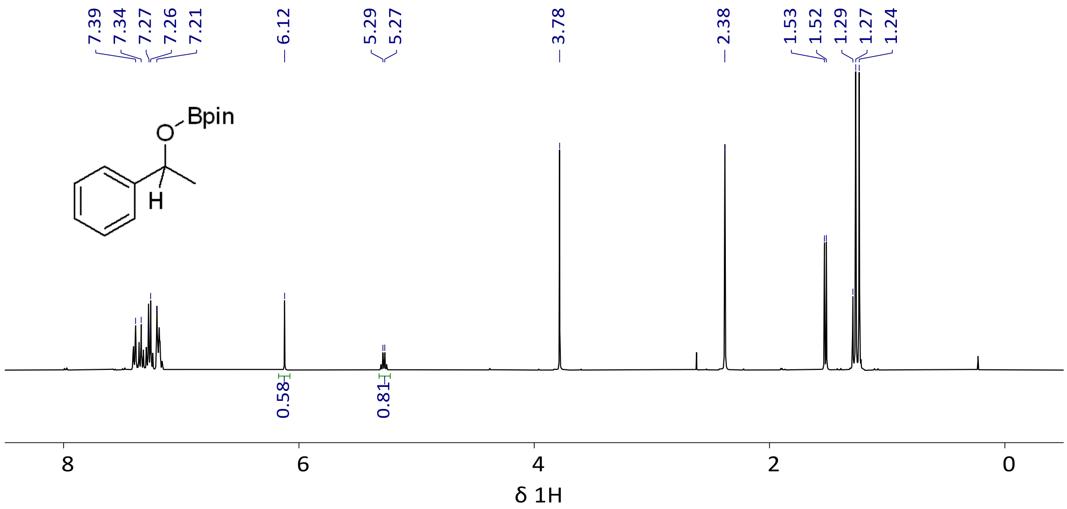

2b (400 MHz, CDCl$_3$, 300 K): δ (ppm) 7.45-7.19 (m, 10H, Ar-*H*), 6.22 (s, 1H, C-*H*), 1.22 (s, 12H, Bpin-Me).[6]

Yield (71%) calculated against 1,3,5-trimethoxybenzene (19.3 mol%): δ 6.12 (s, 3H, Ar-*H*), 3.77 (s, 9H, O-C*H*$_3$).

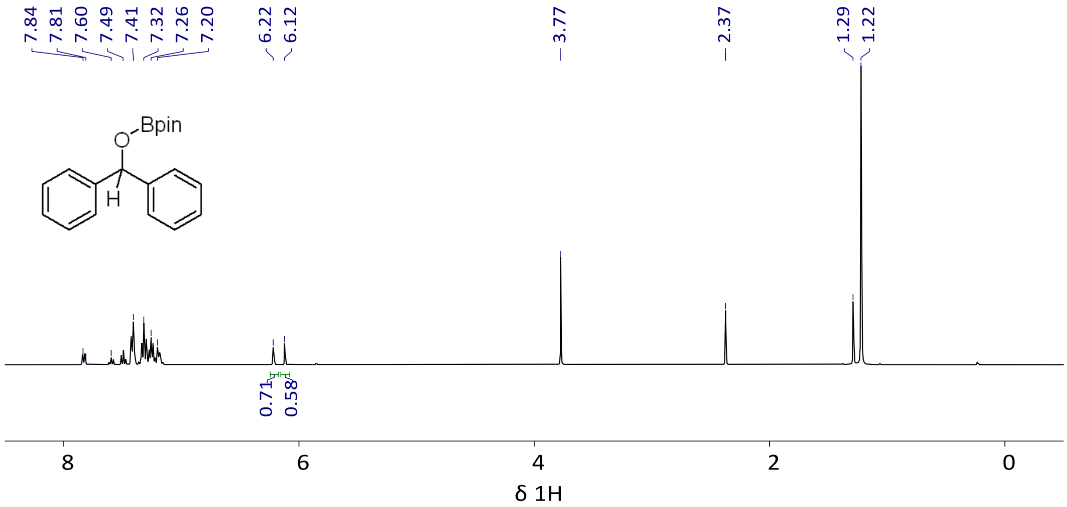

2c (400 MHz, CDCl$_3$, 300 K): δ (ppm) 7.44-7.41 (m, 1H, Ar-*H*), 7.19-7.14 (m, 2H, Ar-*H*), 7.11-7.09 (m, 1H, Ar-*H*), 5.23 (t, $^3J_{H-H}$ = 4.8 Hz, C-*H*), 2.85-2.82 (m, 1H, C*H*$_2$), 2.77-2.73 (m, 1H, C*H*$_2$), 2.11-2.04 (m, 1H, C*H*$_2$), 1.83-1.74 (m, 1H, C*H*$_2$), 1.33 (s, 6H, Bpin-Me), 1.32 (2, 6H, Bpin-Me).[7]

Yield (37%) calculated against 1,3,5-trimethoxybenzene (19.3 mol%): δ 6.12 (s, 3H, Ar-*H*), 3.80 (s, 9H, O-C*H*$_3$).

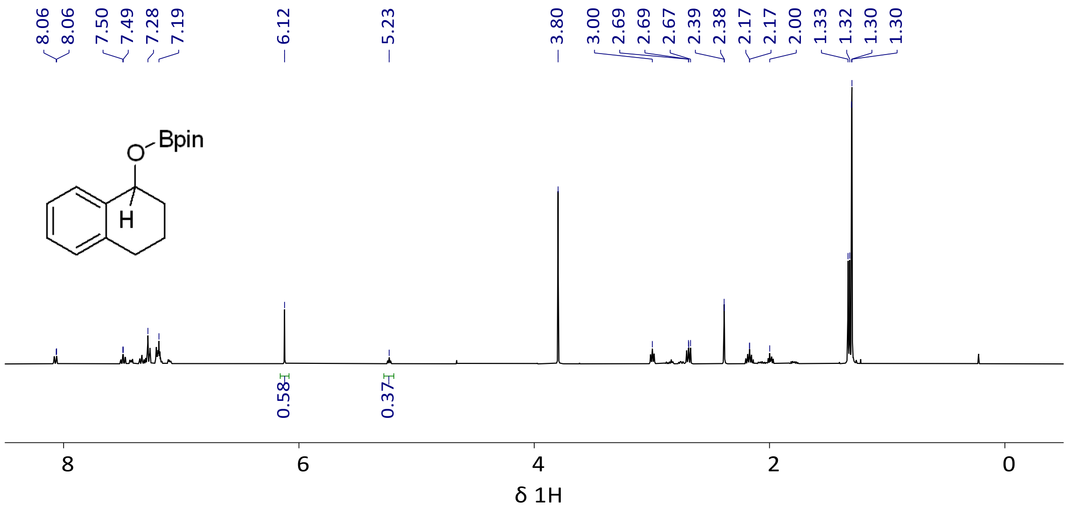

2d (400 MHz, $CDCl_3$, 300 K): δ (ppm) 7.49-7.17 (m, 5H, Ar-*H*), 5.28 (s, 1H, C-*H*), 1.77 (s, 3H, $C(CH_3)_2$), 1.71 (s, 3H, $C(CH_3)_2$), 1.27 (s, 6H, Bpin-Me), 1.22 (2, 6H, Bpin-Me).

Yield (61%) calculated against 1,3,5-trimethoxybenzene (19.3 mol%): δ 6.12 (s, 3H, Ar-*H*), 3.80 (s, 9H, O-CH_3).

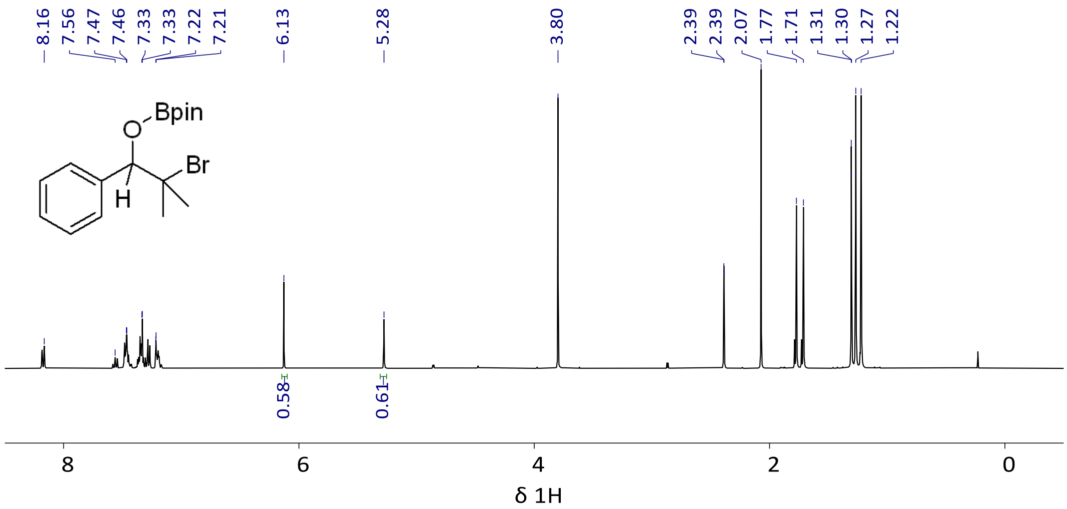

2e Major diastereomer (500 MHz, $CDCl_3$, 300 K): δ (ppm) 7.42-7.14 (m, 5H, Ar-*H*), 5.12 (d, 1H, $^3J_{H-H}$ = 7.4 Hz), 4.31-4.26 (m, 1H, C*H*Me), 1.54 (d, 3H, $^3J_{H-H}$ = 6.9 Hz, CH_3), 1.27 (s, 6H, Bpin-Me), 1.24 (2, 6H, Bpin-Me).

Minor diasteromer (500 MHz, $CDCl_3$, 300 K): δ (ppm) 7.42-7.14 (m, 5H, Ar-*H*), 5.40 (d, 1H, $^3J_{H-H}$ = 4.2 Hz), 4.33 (dp, 1H, $^3J_{H-H}$ = 4.2, 6.8 Hz, C*H*Me), 1.60 (d, 3H, $^3J_{H-H}$ = 6.8 Hz, CH_3), 1.29 (s, 6H, Bpin-Me), 1.25 (2, 6H, Bpin-Me).

Combined yield (47%) calculated against 1,3,5-trimethoxybenzene (19.3 mol%): δ 6.12 (s, 3H, Ar-*H*), 3.80 (s, 9H, O-CH_3).

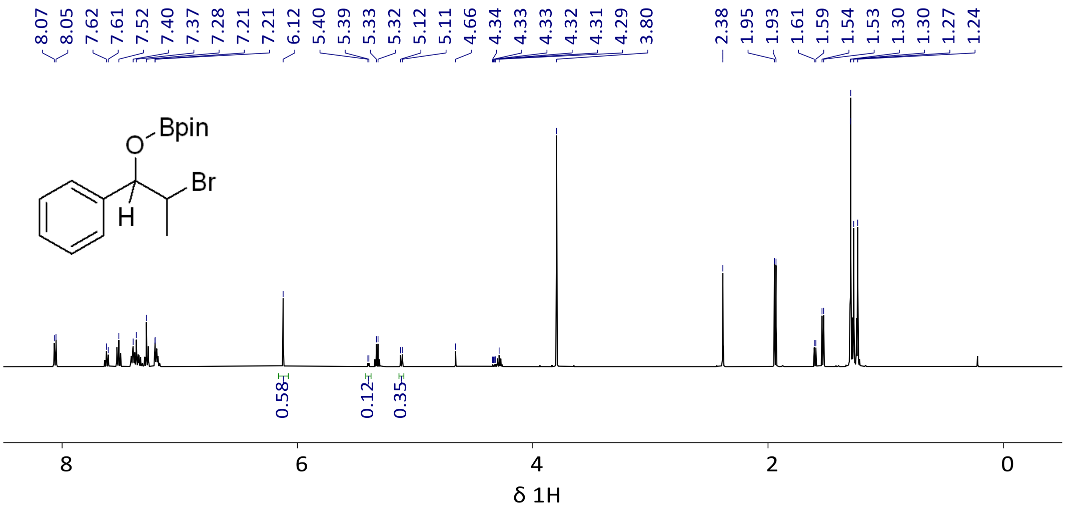

2f (500 MHz, $CDCl_3$, 300 K): δ (ppm) 7.64 (d, 2H, $^3J_{H\text{-}H}$ = 8.0 Hz, Ar-<u>*H*</u>), 7.54 (d, 2H, $^3J_{H\text{-}H}$ = 8.0 Hz, Ar-<u>*H*</u>), 5.36 (q, 1H, $^3J_{H\text{-}H}$ = 6.5 Hz, C-<u>*H*</u>), 1.56 (d, 3H, $^3J_{H\text{-}H}$ = 6.5 Hz, Me), 1.31 (s, 6H, Bpin-Me), 1.28 (2, 6H, Bpin-Me).[5]

Yield (39%) calculated against 1,3,5-trimethoxybenzene (19.3 mol%): δ 6.12 (s, 3H, Ar-<u>*H*</u>), 3.80 (s, 9H, O-C<u>*H*</u>$_3$).

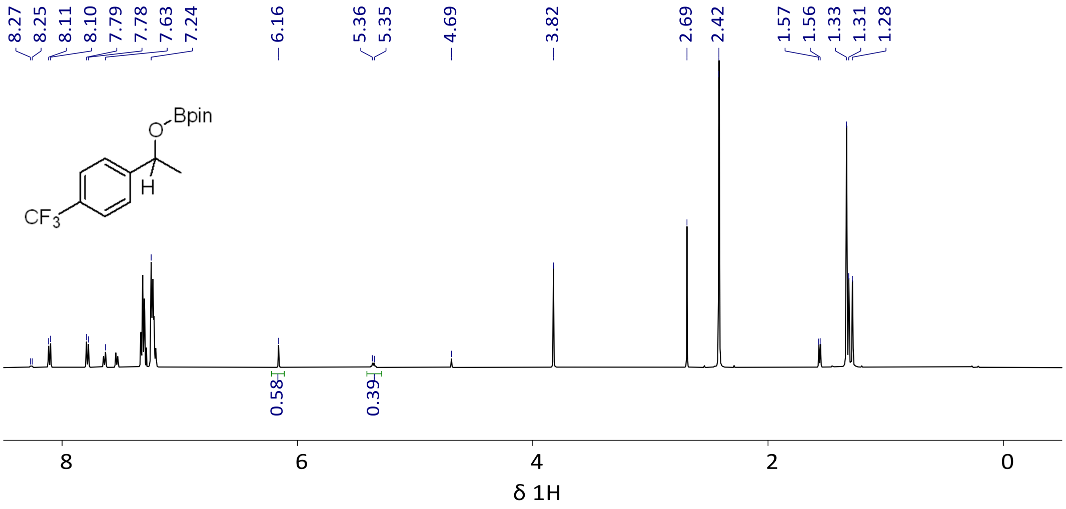

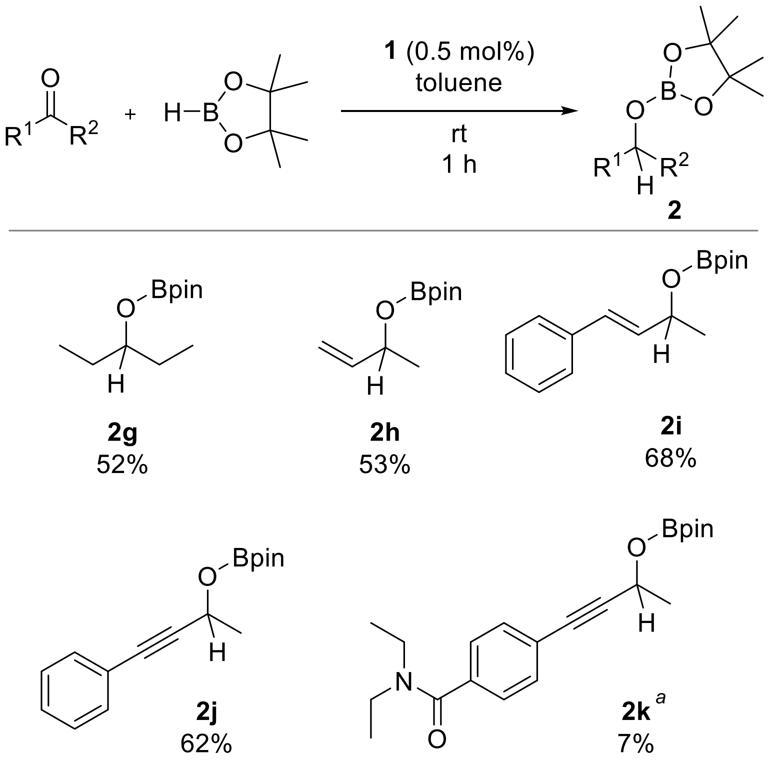

*For **2h** and **2i** analytical data were in accordance with the literature.*[8,9] *Products **2g**, **2j** and **2k** are previously unreported compounds.* [a] *Additional toluene (200 µL) was added to dissolve the substrate.*

2g (400 MHz, $CDCl_3$, 300 K): δ (ppm) 3.90 (tt, 1H, $^3J_{H\text{-}H}$ = 5.1, 7.2 Hz, C*H̲*^{i}Pr$_2$), 1.54-1.44 (m, 4H, C*H̲*$_2$ ×2), 0.92 (t, 6H, $^3J_{H\text{-}H}$ = 7.4 Hz, C*H̲*$_3$ ×2), 1.27 (s, 12H, Bpin).

Yield (52%) calculated against 1,3,5-trimethoxybenzene (19.3 mol%): δ 6.12 (s, 3H, Ar-*H̲*), 3.80 (s, 9H, O-C*H̲*$_3$).

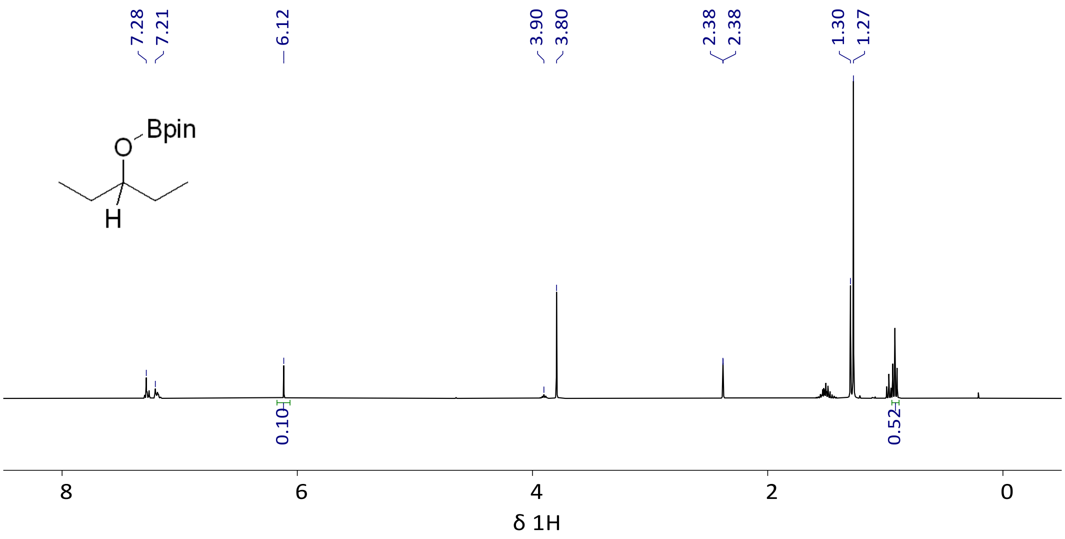

2h (600 MHz, $CDCl_3$, 300 K): δ (ppm) 5.93-5.87 (m, 1H, H_2C=C*H̲*), 5.25-5.21 (m, 1H, *H̲*$_2$C=CH), 5.07-5.04 (m, 1H, *H̲*$_2$C=CH), 4.74-4.69 (m, 1H C*H̲*O), 1.87-1.86 (m, 1H, C*H̲*$_3$).[8]

Yield (53%) calculated against 1,3,5-trimethoxybenzene (19.3 mol%): δ 6.12 (s, 3H, Ar-*H̲*), 3.80 (s, 9H, O-C*H̲*$_3$).

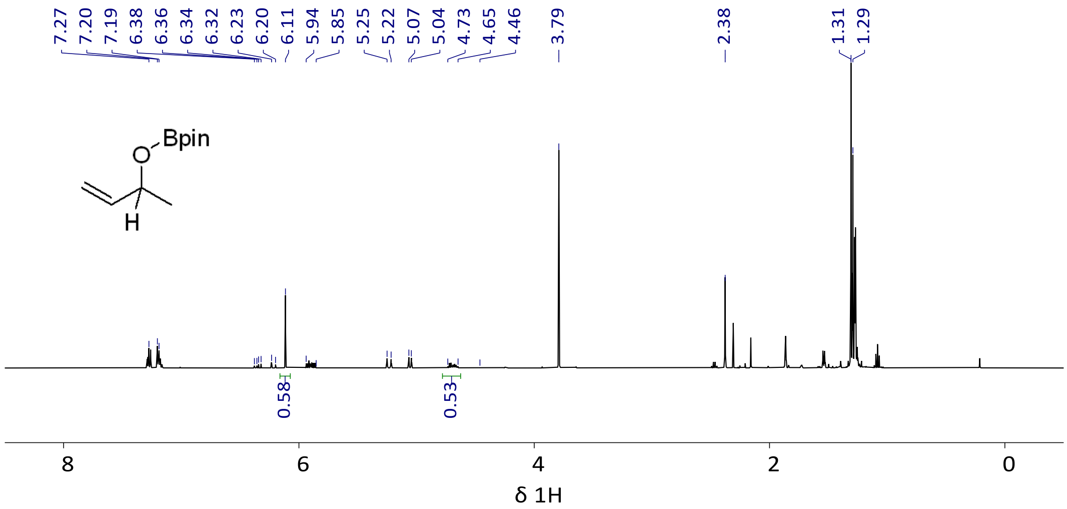

2i (600 MHz, $CDCl_3$, 300 K): δ (ppm) 6.63 (d, 1H, $^{3}J_{H-H}$ = 16.0 Hz, PhC<u>*H*</u>=CH), 6.28 (dd, 1H, $^{3}J_{H-H}$ = 5.8, 16.0 Hz, PhCH=C<u>*H*</u>), 4.91-4.87 (m, 1H, C-<u>*H*</u>), 1.42 (d, 3H, $^{3}J_{H-H}$ = 6.5 Hz, Me), 1.31 (s, 6H, Bpin-Me), 1.30 (2, 6H, Bpin-Me).[9]

Yield (68%) calculated against 1,3,5-trimethoxybenzene (19.3 mol%): δ 6.12 (s, 3H, Ar-<u>*H*</u>), 3.80 (s, 9H, O-C<u>H_3</u>).

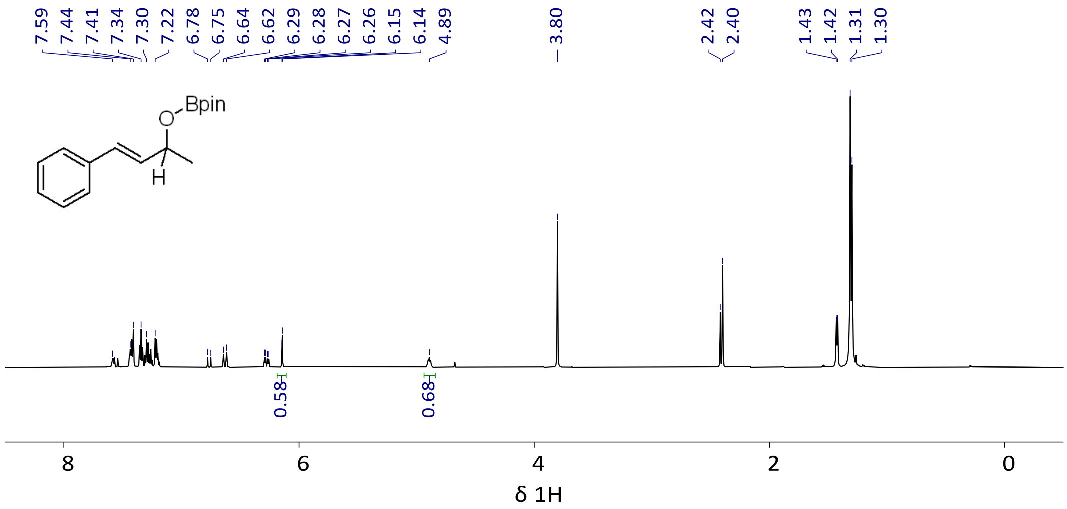

2j (400 MHz, $CDCl_3$, 300 K): δ (ppm) 7.46-7.42 (m, 5H, Ar-<u>*H*</u>), 5.10, (q, 1H, $^{3}J_{H-H}$ = 6.7 Hz, C-<u>*H*</u>), 1.58 (d, 3H, $^{3}J_{H-H}$ = 6.7 Hz, C<u>H_3</u>), 1.30 (s, 12H, Bpin).

Yield (62%) calculated against 1,3,5-trimethoxybenzene (19.3 mol%): δ 6.12 (s, 3H, Ar-<u>*H*</u>), 3.80 (s, 9H, O-C<u>H_3</u>).

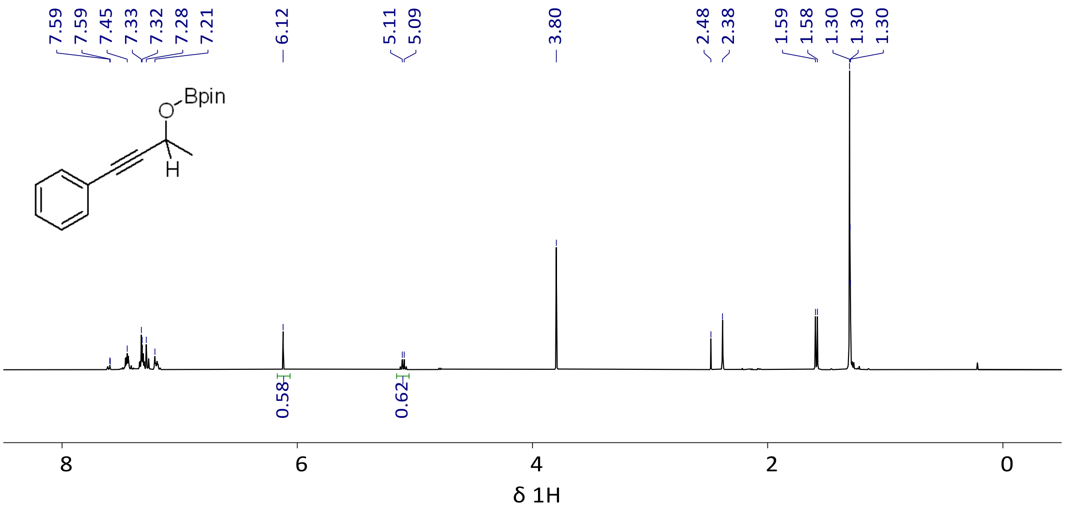

2k The crude reaction mixture showed a complex mixture of products but a small amount of **2k** was identified from characteristic resonances assigned below by comparison with product **2j**.

(400 MHz, $CDCl_3$, 300 K): δ (ppm) 5.10, (q, 1H, $^3J_{H-H}$ = 6.6 Hz, C-<u>*H*</u>), 1.59 (d, 3H, $^3J_{H-H}$ = 6.6 Hz, C<u>*H*</u>$_3$), 1.30 (s, 12H, Bpin).

Yield (7%) calculated against 1,3,5-trimethoxybenzene (19.3 mol%): δ 6.12 (s, 3H, Ar-<u>*H*</u>), 3.80 (s, 9H, O-C<u>*H*</u>$_3$).

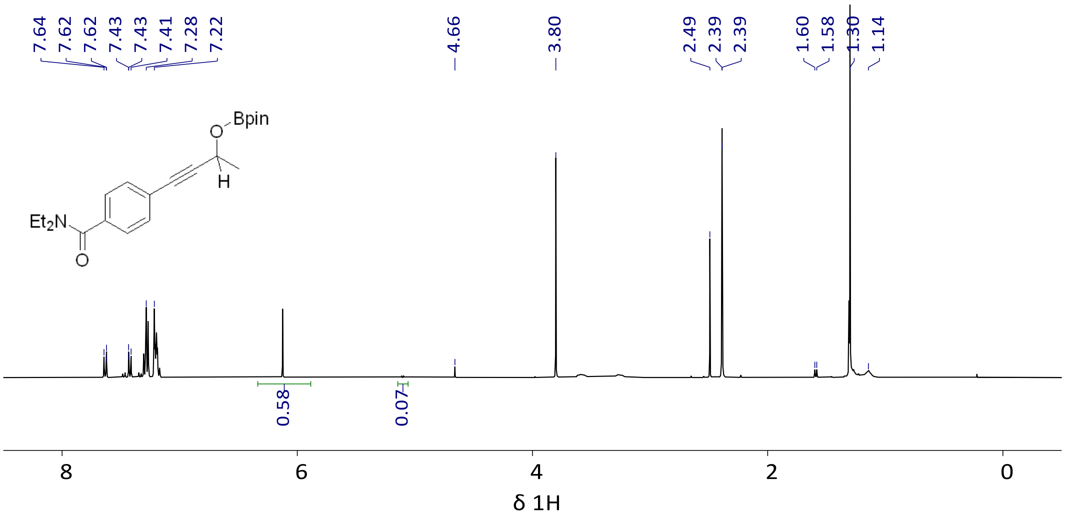

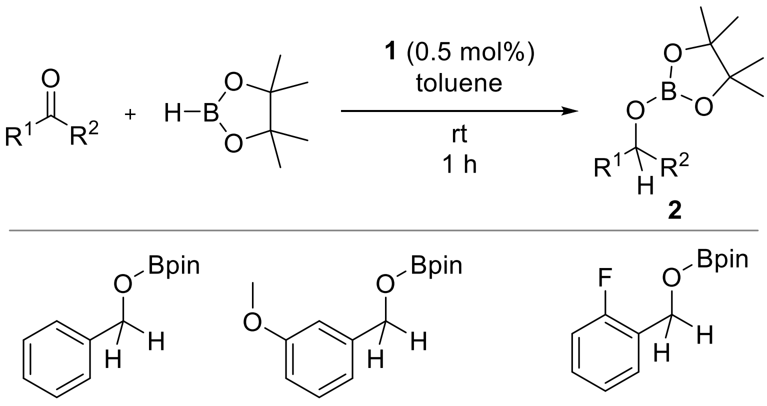

2l >99% **2m** >99% **2n** >99%

NMR data were in accordance with the literature.[10]

2l (400 MHz, $CDCl_3$, 300 K): δ (ppm) 7.27-7.22 (m, 4H), 7.19-7.15 (m, 1H) 4.84 (s, 2H), 1.18 (s, 12H).[10]

2m (400 MHz, $CDCl_3$, 300 K): δ (ppm) 7.28 (d, 2H, $^3J_{H-H}$ = 8.6 Hz), 6.86 (d, 2H, $^3J_{H-H}$ = 8.6 Hz), 4.85 (s, 2H), 3.79 (s, 3H), 1.26 (s, 12H).[10]

2n (400 MHz, $CDCl_3$, 300 K): δ (ppm) 7.50-7.43 (m, 1H), 7.30-7.01 (m, 3H), 5.03 (s, 2H), 1.30 (s, 12H).[10]

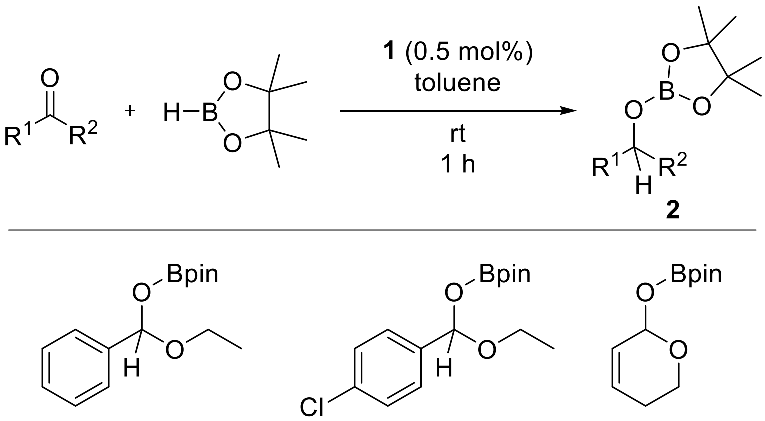

0% 0% 0%

The hydroboration of esters was unsuccessful using our procedure.

5 Stoichiometric Reactivity

Stoichiometric Reaction of HBpin and Silyl Anion 1

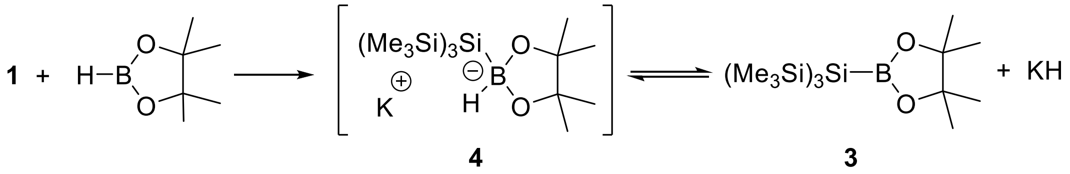

Poor solubility in C_6D_6

In a typical reaction anion **1** (14 mg, 0.07 mmol) was dissolved inTHF-d_8 or C_6D_6 (0.6 mL). Pinacolborane (9 µL, 0.07 mmol) was added at room temperature which resulted in the formation of a gel like solid.

In both solvents the ^{1}H NMR gave broad, intractable signals. In C_6D_6, a single ^{11}B resonance was observed which is assigned to silyl boronic ester **3** in accordance with the literature.[11]

^{11}B NMR (160 MHz, C_6D_6, 300 K): δ (ppm) 37.3.

In THF-d_8 a mixture of boronic ester **3**[11] and an unidentified BH_3 adduct were observed, along with a new resonance which is tentatively assigned to boronate complex **4**.

^{11}B NMR (160 MHz, C_6D_6, 300 K): δ (ppm) 37.3 (**3**), 8.4 (**4**), −45.4 (BH_3 adduct).

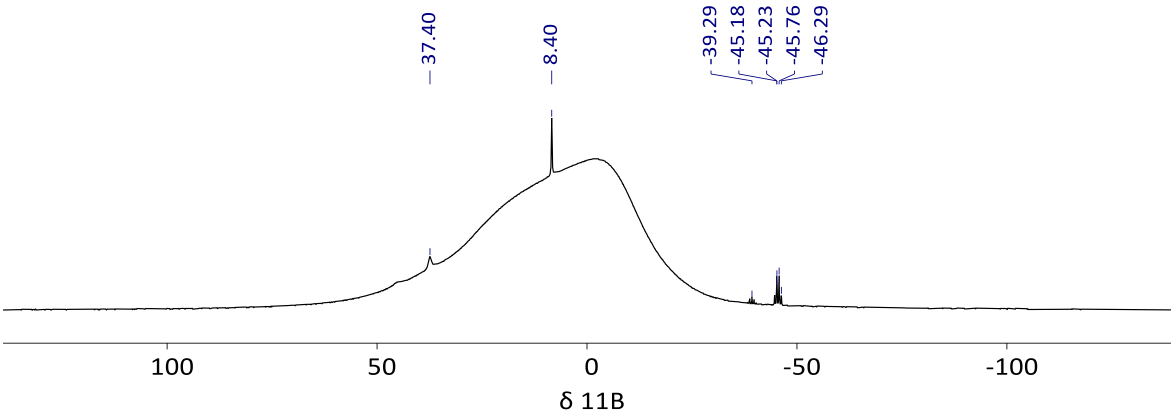

Preparation of Silyl Boronic Ester 3 *via* Boron-ate 4

Silyl anion **1** (99 mg, 0.25 mmol) was dissolved in hexane and HBpin (36 µL, 0.25 mmol) was added dropwise at room temperature giving a colourless solution with a gel-like suspension which was stirred at room temperature for 16 hours. Filtration of the reaction mixture through a silica plug afforded crude silyl boronic ester **3** quantitatively. NMR data were in accordance with the literature.[11]

^{1}H NMR (500 MHz, C_6D_6, 300 K): δ (ppm) 1.19 (s, 12H, Bpin), 0.18 (s, 12H, $Si(SiMe_3)_3$).

^{11}B NMR (160 MHz, C_6D_6, 300 K): δ (ppm) 37.3.

6 Attempted Hydroboration of Alkynes

Test reactions were carried out in order to determine the activity of silyl anion **1** in the hydroboration of alkynes.

As expected, the hydroboration of phenylacetylene did not occur at room temperature in the presence of **1**. However, when the reaction mixture was heated to 60 °C for two hours with 10 mol% catalyst loading, trace yields of the vinyl boronic ester were observed. Heating the reaction mixture to 110 °C gave 74% yield in two hours (entry 4), comparable activity to that reported for aluminum hydrides.[12–14]

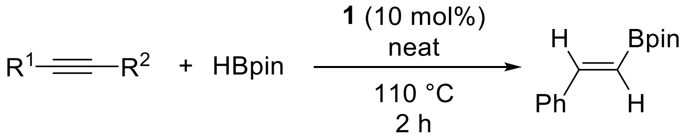

Entry	Catalyst Loading (mol%)	Temperature (°C)	Time (h)	Conversion (%)
**1**	10	rt	0.5	0
**2**	10	60	2	< 3
**3**	5	110	1	17
**4**	10	110	2	74

0.5 mmol substrate, 0.55 mmol HBpin, neat.

Initially the silyl anion catalyzed hydroboration of alkynes was tested with a small number of substrates (Scheme S1). The highest yield was found for phenylacetylene but all four substrates showed some conversion. Terminal alkynes were 100% selective for the linear product.

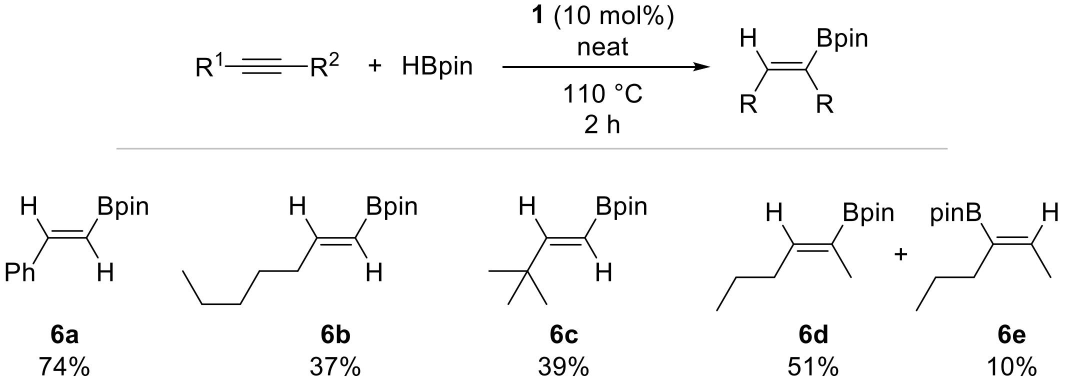

Scheme S1 *– Substrates in the silyl anion catalyzed hydrboration of actylenes. Reaction conditions: 0.5 mmol substrate, 0.55 mmol HBpin, no solvent, 110 °C, 2 hours. NMR yield measured against trimethoxybenzene as an internal standard.*

The results for the hydroboration using **1** as a catalyst were largely unreliable, with yields varying from 20% to 80% under the same conditions for the same substrate. Furthermore, BH_3 was observed in the stoichiometric reaction between HBpin and **1** when it was carried out in THF, as mentioned in the main text. This is likely exacerbated by the high reaction temperatures and long reaction times.

The catalyst free hydroboration of alkynes by HBpin was reported with the initial publication of the preparation of HBpin,[15] presumably due to contamination by the BH_3 which was used to synthesize the HBpin. Recently the BH_3 catalyzed hydroboration of alkynes has also been reported by some of us.[16]

Given the unreliable results and the likelihood that the catalysis is performed by BH_3, the hydroboration of alkynes was not further pursued.

^{1}H NMR Data for Alkyne Hydroboration Products

NMR data were in accordance with the literature.[14,17]

6a (600 MHz, $CDCl_3$, 300 K): δ (ppm) 7.52 – 7.50 (m, 2H, Ar-*H*), 7.44 (d, 1H $^{3}J_{H\text{-}H}$ = 18.4 Hz, C-*H*), 7.38 – 7.32 (m, 3H, Ar-*H*), 6.20 (d, 1H, $^{3}J_{H\text{-}H}$ = 18.4 Hz, C-*H*), 1.35 (s, 12H, Bpin).[14]

6b (500 MHz, $CDCl_3$, 300 K): δ (ppm) 6.64 (dt, 1H, $^{3}J_{H\text{-}H}$ = 18.0, 6.4 Hz, C-*H*), 5.4 (d, 1H, $^{3}J_{H\text{-}H}$ = 18.0, C-*H*), 1.27 (s, 12H, Bpin).[17]

6c (500 MHz, $CDCl_3$, 300 K): δ (ppm) 6.65 (d, 1H, $^{3}J_{H\text{-}H}$ = 18.3 Hz, C-*H*), 5.36 (d, 1H, $^{3}J_{H\text{-}H}$ = 18.3 Hz, C-*H*), 1.28 (s, 12H), 1.03 (s, 9H).[14]

6d (500 MHz, $CDCl_3$, 300 K): δ (ppm) 6.31 (t, 1H, $^{3}J_{H\text{-}H}$ = 7.1 Hz, C-*H*) 2.11 (q, 4H, $^{3}J_{H\text{-}H}$ = 7.3), 1.72 (d, 3H, $^{3}J_{H\text{-}H}$ = 7.0 Hz), 1.69 (s, 3H), 1.48-1.40 (m, 2H), 1.27 (s, 12H), 0.93 (t, 3H $^{3}J_{H\text{-}H}$ = 7.3Hz).[14]

6e (500 MHz, $CDCl_3$, 300 K): δ (ppm) 6.43 (q, 1H, $^{3}J_{H\text{-}H}$ = 6.7 Hz), 1.74 (d, 3H, $^{3}J_{H\text{-}H}$ = 6.7 Hz), 1.40-1.35 (m, 2H), 1.24 (s, 12H), 0.91 (t, 3H, $^{3}J_{H\text{-}H}$ = 7.6 Hz).[14]

7 Substrate Scope NMR Spectra

8 References

[1] G. R. Fulmer, A. J. M. Miller, N. H. Sherden, H. E. Gottlieb, A. Nudelman, B. M. Stoltz, J. E. Bercaw, K. I. Goldberg, *Organometallics* **2010**, *29*, 2176–2179.
[2] C. Marschner, *Eur. J. Inorg. Chem.* **1998**, *1998*, 221–226.
[3] M. W. Stanford, J. I. Schweizer, M. Menche, G. S. Nichol, M. C. Holthausen, M. J. Cowley, *Angew. Chem. Int. Ed.* **2019**, *58*, 1329–1333.
[4] A. H. Cowley, E. a. V. Ebsworth, S. K. Mehrotra, D. W. H. Rankin, M. D. Walkinshaw, *J. Chem. Soc. Chem. Commun.* **1982**, 1099–1100.
[5] S. Khoo, J. Cao, F. Ng, C.-W. So, *Inorg. Chem.* **2018**, *57*, 12452–12455.
[6] P. K. Verma, S. A. S., K. Geetharani, *Org. Lett.* **2018**, *20*, 7840–7845.
[7] S. Chen, D. Yan, M. Xue, Y. Hong, Y. Yao, Q. Shen, *Org. Lett.* **2017**, *19*, 3382–3385.
[8] M. Ma, J. Li, Q. Xiao, M. Luo, *Asymmetric Diimine Monovalent Magnesium Compound, and Preparation Method and Application Thereof in Epoxy Silane Hydroboration*, **2018**, CN108569984 (A).
[9] V. L. Weidner, C. J. Barger, M. Delferro, T. L. Lohr, T. J. Marks, *ACS Catal.* **2017**, *7*, 1244–1247.
[10] H. Stachowiak, J. Kaźmierczak, K. Kuciński, G. Hreczycho, *Green Chem.* **2018**, *20*, 1738–1742.
[11] E. Yamamoto, R. Shishido, T. Seki, H. Ito, *Organometallics* **2017**, *36*, 3019–3022.

[12] Z. Yang, M. Zhong, X. Ma, S. De, C. Anusha, P. Parameswaran, H. W. Roesky, *Angew. Chem. Int. Ed.* **2015**, *54*, 10225–10229.
[13] D. Franz, L. Sirtl, A. Pöthig, S. Inoue, *Z. Für Anorg. Allg. Chem.* **2016**, *642*, 1245–1250.
[14] A. Bismuto, S. P. Thomas, M. J. Cowley, *Angew. Chem. Int. Ed.* **2016**, *55*, 15356–15359.
[15] C. E. Tucker, J. Davidson, P. Knochel, *J. Org. Chem.* **1992**, *57*, 3482–3485.
[16] N. W. J. Ang, C. S. Buettner, S. Docherty, A. Bismuto, J. R. Carney, J. H. Docherty, M. J. Cowley, S. P. Thomas, *Synthesis* **2018**, *50*, 803–808.
[17] D. P. Ojha, K. R. Prabhu, *Org. Lett.* **2016**, *18*, 432–435.
